# The Function, Regulation and Mechanism of Programmed Cell Death of Macrophages in Atherosclerosis

**DOI:** 10.3389/fcell.2021.809516

**Published:** 2022-01-11

**Authors:** Chang Liu, Zecheng Jiang, Zhongjie Pan, Liang Yang

**Affiliations:** ^1^ Department of Pharmacology, School of Medicine, Nankai University, Tianjin, China; ^2^ Tianjin Union Medical Center, Tianjin, China

**Keywords:** atherosclerosis, macrophage, programmed cell death, apoptosis, autophagy, pyroptosis, ferroptosis, necroptosis

## Abstract

Atherosclerosis is a chronic progressive inflammatory vascular disease, which is an important pathological basis for inducing a variety of cardio-cerebrovascular diseases. As a kind of inflammatory cells, macrophages are the most abundant immune cells in atherosclerotic plaques and participate in the whole process of atherosclerosis and are the most abundant immune cells in atherosclerotic plaques. Recent studies have shown that programmed cell death plays a critical role in the progression of many diseases. At present, it is generally believed that the programmed death of macrophages can affect the development and stability of atherosclerotic vulnerable plaques, and the intervention of macrophage death may become the target of atherosclerotic therapy. This article reviews the role of macrophage programmed cell death in the progression of atherosclerosis and the latest therapeutic strategies targeting macrophage death within plaques.

## Introduction

Atherosclerosis is a chronic inflammatory vascular disease with complex pathogenesis, which is the pathological basis of a variety of cardiovascular and cerebrovascular diseases and has always been the focus of medical researchers. Atherosclerosis, especially ruptured or eroded plaque and subsequent acute cardio-cerebrovascular complications, remains the leading cause of mortality worldwide (Roth et al., 2017). The major immune cell in atherosclerotic lesions is the macrophage, the origin of which is circulating monocytes. Within plaques, macrophages participate in the progression of the atherosclerotic lesion via uptake of oxidized low-density lipoprotein (ox-LDL) and ensuing foam cell formation. Moreover, macrophages can create broad-spectrum cytokines and chemokines that cause atherosclerosis to influence plaque stability, and inflammatory responses of macrophages are also a driving force for atherosclerotic progression and plaque growth (Shashkin et al., 2005; Shimada, 2009).

Macrophage proliferation, aggregation, aging, and death have an impact on the occurrence and development of atherosclerosis. Therefore, these aspects can also be used as potential directions of anti-atherosclerotic therapy. Among them, macrophage programmed cell death plays a particularly important role in atherosclerosis. Within advanced atherosclerotic plaques, macrophages occupied nearly half of the dead cell population. The aggregation and death of macrophages may promote the formation and enlargement of lipid necrotic core and the instability of plaques (Lutgens et al., 1999). Previous studies have shown that macrophages in plaques may undergo several types of programmed death, including apoptosis, autophagy, pyroptosis, ferroptosis and necroptosis. Different macrophage death patterns and occur stages will have a promotive or inhibitory effect on atherosclerosis. Based on the current knowledge of macrophages and atherosclerosis, diverse drugs can be designed to mitigate the progress of atherosclerosis and stabilize vulnerable plaques. Next, this review will concentrate on the research progress of various programmed death modes and targeted therapy of macrophages in atherosclerotic plaques.

### Effect of Macrophage Apoptosis on Atherosclerosis

#### Apoptosis and Its Mechanism

Apoptosis is a form of programmed cell death, which is an active process of automatic termination of life determined by genes, with strict regulatory signal pathways (Fuchs and Steller, 2011). Apoptosis plays a key role in the normal ontogeny of multicellular organisms, the maintenance of self-stable balance and resistance to the interference of various external factors. Different from cell necrosis, apoptosis has the characteristic nuclear condensation in morphology, and the chromosomes are cut into 180bp-200 bp-sized fragments in nucleosomes. Then, the cells shrink and finally form apoptotic bodies. In the process of apoptosis, the phosphatidylserine (PtdSer) on the inside of the cell membrane is turned out to the surface of the membrane, but the structure of the cell membrane is still intact. Apoptotic bodies can be quickly swallowed by the surrounding professional or non-professional phagocytes, so it will not cause the surrounding inflammatory response (Jacobson et al., 1997).

Apoptosis mainly includes exogenous pathway and endogenous pathway. Exogenous pathway, also known as death receptor pathway, activates the caspase family of aspartate proteolytic enzymes and induces cell apoptosis mediated by transmembrane receptors. The endogenous pathway, also known as the mitochondrial pathway, stimulates the direct production of intracellular signals, causes changes in the structure of mitochondrial membrane, releases pro-apoptotic substances, and induces cell apoptosis (Peter, 2011).

#### Mechanism and Role of Macrophage Apoptosis in Different Stages of Atherosclerosis

**FIGURE 1:**
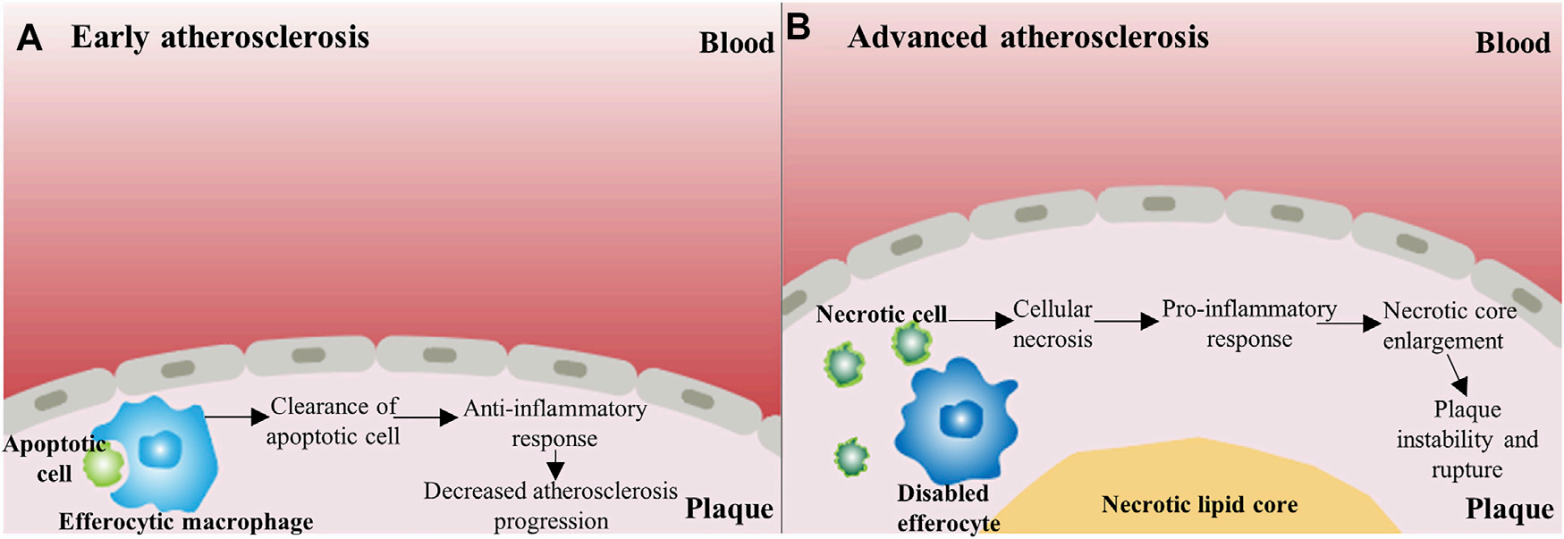
The dual role of macrophage apoptosis in early and advanced atherosclerosis **(A)** In early atherosclerosis, efferocytosis is efficient, and apoptotic cells are rapidly cleared by efferocytes, which can trigger a positive anti-inflammatory response, then resulting in decreased atherosclerosis progression **(** **B)** In advanced atherosclerosis, disabled efferocytes cannot function properly. Subsequently, the accumulation of apoptotic cells results in secondary necrosis and an inflammatory response ensues. These phenomena may further increase the risk of plaque rupture.

In the area of atherosclerotic lesions, the macrophages with apoptosis account for the majority of the total apoptotic cells. The apoptosis of macrophages can ensue during all atherosclerotic stages and affect early lesion formation, plaque progression, and plaque stability. Previous studies have shown that there is a duality in the regulation of macrophage apoptosis on the development of atherosclerosis. Loss of JNK1, the pro-apoptotic effector, in hematopoietic cells protected macrophages from apoptosis and this accelerated early atherosclerosis in Ldlr^−/−^ mice (Babaev et al., 2016). Myocardin-related transcription factor A (MRTF-A) was highly expressed in macrophages of human carotid atherosclerotic plaque. Experiments both *in vivo* and *in vitro* reveal that MRTF-A can elevate proliferation and attenuate apoptosis of macrophages and overexpression MRTF-A in monocytes aggravated atherosclerosis in ApoE knockout mice (An et al., 2019). Consistent with these findings, Ldlr^−/−^ mice showed an increase in macrophage apoptosis and an inhibition of early atherosclerosis under the deletion of apoptosis inhibitor AIM (Spα/Api6) (Arai et al., 2005). These results support the concept that macrophage apoptosis is a negative regulator of atherosclerotic plaque development. It seems that increased macrophage apoptosis is related to diminished cellularity within the lesion area and decreased lesion progression in the early stage of atherosclerosis. However, other studies confirm that macrophage apoptosis positively regulates the development of atherosclerosis. Shan Shu et al. found that the expression of monocyte chemotactic protein-induced protein 1 (MCPIP1) can be promoted by Angiotensin II (Ang II) through an AMPK/p38 MAPK-dependent pathway. Increased MCPIP1 expression triggered endoplasmic reticulum (ER) stress to induce macrophage apoptosis and additionally participated in the formation of rupture-prone plaques (Shu et al., 2019). Overexpression of miR-10b in ApoE^−/−^ mice mitigated plaque macrophage apoptosis, reduced late plaque size and increased plaque stability, but had no effect on early plaque formation (Wang et al., 2018). In addition, homocysteine (Hcy), via upregulation of endoplasmic reticulum oxidoreductase 1*α* (Ero1*α*) expression, activated ER stress-dependent macrophage apoptosis to expedite vulnerable plaque formation in advanced atherosclerosis (Zhang et al., 2021). It has been proposed that continuous apoptosis of macrophages in stable plaques contributes to the necrotic core expansion and fragility to rupture (Isner et al., 1995; Mallat et al., 1997). According to previous studies, the dual role of macrophage apoptosis in regulating atherosclerosis development depends on the stage of atherosclerotic plaque progression. Macrophage apoptosis decreases lesion cellularity and progression in early lesions but promotes plaque necrosis in more-advanced lesions. This may be due to the deficient efferocytotic removal of apoptotic cells and foam cells in advanced atherosclerotic plaques. In normal physiology, apoptotic cells in plaques can release “find me” signal molecules to effectively recruit phagocytes, and express “eat me” signals on the cell membrane to promote efferocytosis. For example, PtdSer of apoptotic cell membrane structure is a strong “eat me” signal. A variety of phagocytic receptors, such as brain-specific angiogenesis inhibitor one and TIM receptor family, can bind to it to facilitate efferocytosis (Elliott et al., 2017). In the context of this circumstance, the efficiency of apoptosis is very high, and both professional and non-professional phagocytes are able to remove apoptotic bodies rapidly. More importantly, the clearance of apoptotic cells can trigger a positive anti-inflammatory response in efferocytes (Fadok et al., 1998). Therefore, in early plaque, macrophage apoptosis can reduce inflammation and inhibit plaque progression through efficient efferocytosis (Figure 1A). However, in progressive plaques, despite the existence of a large number of “find me” and “eat me” signals, ox-LDL can block the effective phagocytic receptors on the surface of efferocytes, such as c-Mer tyrosine kinase (MerTK) receptors, and the efficient efferocytosis cannot play a full role. Gene knockout of MerTK receptors leads to plaque apoptotic cell accumulation and necrotic core expansion (Ait-Oufella et al., 2008). Especially in the inflammatory environment in plaques, the MerTK is cut to form soluble MerTK receptors, which significantly weakens the effective efferocytotic ability of efferocytes (Cai et al., 2017). Hence, in advanced plaques where efferocytosis is not efficient, the accumulation of apoptotic cells results in secondary necrosis and an inflammatory response ensues (Henson et al., 2001). These phenomena may further cause the release of cellular inflammatory contents, boost plaque instability and increase the risk of clinical events of acute atherosclerotic thrombosis (Figure 1B).

#### Pharmacological Intervention of Macrophage Apoptosis in the Treatment of Atherosclerosis

Macrophage apoptosis has a negative regulatory effect on the development of early atherosclerotic plaque, but positively regulates atherosclerotic lesion development in advanced atherosclerotic plaque. So, increasing macrophage apoptosis in early atherosclerotic plaque and reducing macrophage apoptosis in advanced atherosclerotic plaque may be an effective mechanism for the management and treatment of atherosclerosis. Existing drugs such as statins and drugs targeted by angiotensin converting enzyme inhibitors have been shown to regulate apoptosis in atherosclerotic plaques (Zhang et al., 2018). However, the side effects of these drugs are more serious, long-term use will cause muscle soreness, liver function damage and neurotoxicity and so on. In recent years, it has been found that sonodynamic therapy (SDT), as a new non-invasive targeted physiotherapy, has a good therapeutic effect on atherogenesis. Xin Sun et al. found that SDT can induce macrophage apoptosis, reduce the number and lipid content of macrophages, and stabilize atherosclerotic plaques. So it played an active role in delaying the progression of the disease (Sun et al., 2019). Moreover, Viorel Simion et al. showed that the macrophage-specific lncRNA MAARS regulated apoptosis via interacting with HuR which was a critical mediator of transcriptional stability and apoptosis. And MAARS deficiency in macrophages decreased apoptosis and increased their efferocytosis, consequently, reducing atherosclerotic lesion formation by 52% in Ldlr^−/−^ mice. Therefore, either reducing MAARS expression or blocking MAARS–HuR interaction could presumably be effective strategies in limiting macrophage apoptosis in advanced plaques (Simion et al., 2020).

In addition, the treatment of atherosclerosis is mostly related to the enhancement of macrophage efferocytosis. A recent study has shown that synthetic simulated apoptotic cells such as PtdSer encapsulated nanoparticles can be used to enhance efferocytosis (Bagalkot et al., 2016). However, most of the studies on enhancing the role of macrophage efferocytosis to treat atherosclerosis only stay at the basic experimental level, so actively exploring whether these methods can be used in clinical anti-atherosclerosis by enhancing the effect of efferocytosis needs to be further discussed.

### Effect of Macrophage Autophagy on Atherosclerosis

#### Autophagy and Its Occurrence Process

Autophagy is also a form of programmed cell death, also known as type Ⅱ programmed cell death, which participates in the occurrence and development of many diseases. Autophagy refers to the process in which cells use lysosomes or vacuoles to degrade damaged organelles and macromolecular substances under the induction of nutritional deficiency, hypoxia and reactive oxygen species, which can provide raw materials for cell reconstruction, regeneration and repair. It is a compensatory and self-protecting catabolic cellular pathway and a defense and protective mechanism to maintain cell homeostasis (Han et al., 2018). Based on the physiological function and delivery routine to the lysosomal lumen, autophagy is able to be divided into three different forms: macroautophagy, microautophagy and chaperone-mediated autophagy (Yang and Klionsky, 2010). The term autophagy commonly refers to macroautophagy, which is the most prevalent and extensively studied form of autophagy. Autophagy introduced later also refers to macroautophagy. The process of autophagy is that the degradation is wrapped by vesicles with bilayer structure to form autophagosomes, and the outer membrane of the latter then fuses with lysosomal membrane or tonoplast, releasing the encapsulated substance into lysosome or vacuole, and finally hydrolyzing it into small molecular compounds such as amino acids, carbohydrates, fatty acids and nucleotides under the action of a series of hydrolytic enzymes (Levine and Kroemer, 2008). Basal autophagy can remove excess or damaged substances in cells in time, and reuse degradation products, which helps to maintain the normal metabolic function and survival of cells.

#### The Protective Role of Macrophage Autophagy in Atherosclerosis

**FIGURE 2:**
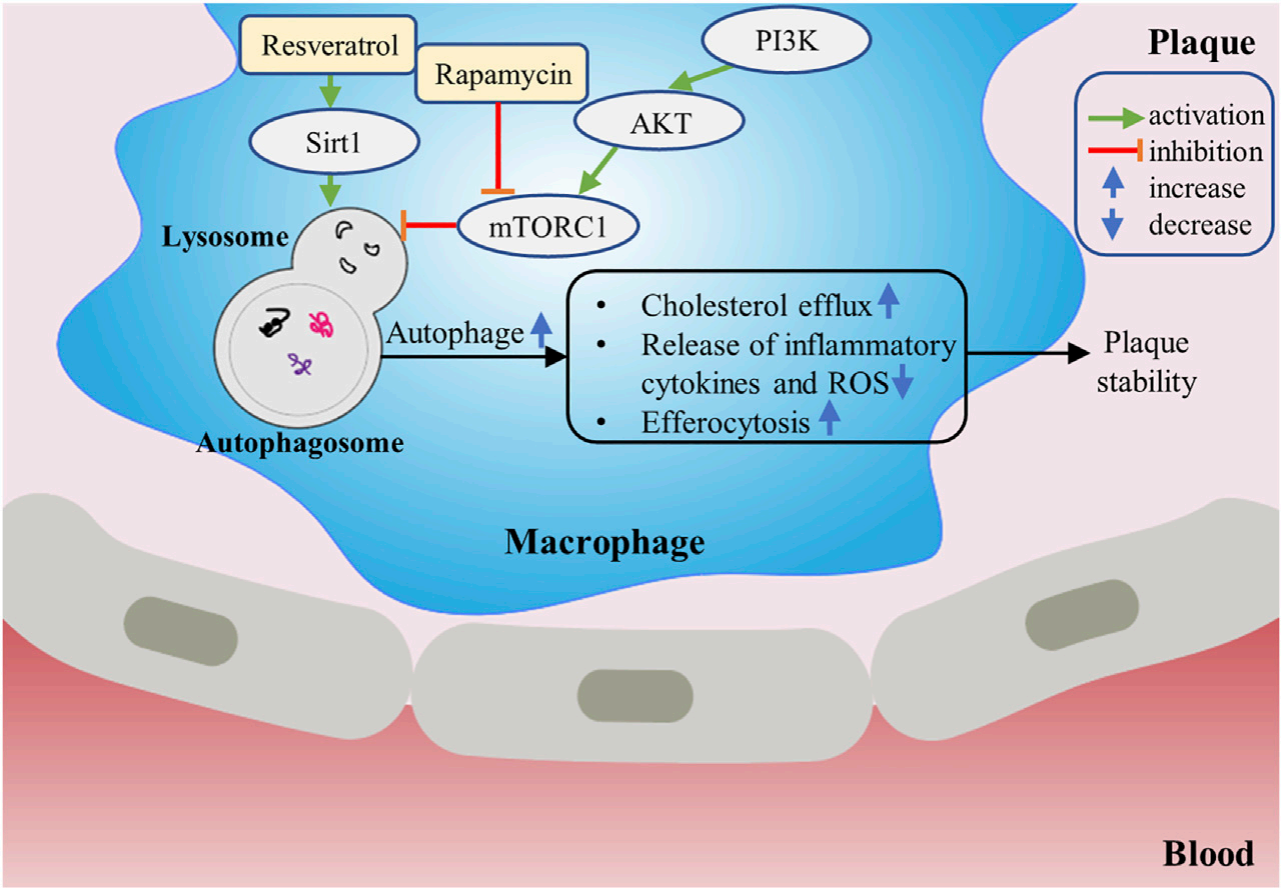
The protective role of macrophage autophagy in atherosclerosis. Drugs such as rapamycin can increase autophagy of macrophages by inhibiting mTORC1. Up-regulation of macrophage autophagy can increase cholesterol efflux, promote efferocytosis, inhibit inflammatory response and suppress oxidative stress, thereby contributing to stabilizing the plaque.

Autophagy is involved in the regulation of cell survival and death during atherosclerosis and runs through the whole process. In the process of plaque formation in mice, autophagic markers mainly colocalized with macrophages and dysfunctional autophagy is a characteristic of plaques (Razani et al., 2012). Studies have found that up-regulation of macrophage autophagy can slow down the progression of atherosclerosis and mitigate the vulnerability of atherosclerotic plaques. Shaohong Fang’s findings indicated that arsenic trioxide (ATO) promoted reactive oxygen species (ROS) induction, which resulted in inhibition of the PI3K/AKT/mTOR pathway, ultimately promoting macrophage autophagy and reducing atherosclerotic lesions at early stages (Fang et al., 2021). The main reason for the foam formation of vascular wall macrophages is the imbalance of cholesterol in and out, and macrophage autophagy can mediate cholesterol efflux and reduce foam cell formation. Shuilong Leng et al. discovered that ursolic acid (UA) enhanced macrophage autophagy and facilitated macrophage cholesterol efflux. Both of these effects are anti-atherosclerotic, resulting in reduced atherogenesis in Ldlr^−/−^ mice fed a Western diet (Leng et al., 2016). Mingxue Zhou et al. found that Shen-Yuan-Dan Capsule (SYDC) treatment can ameliorate the level of blood lipid, reduce the atherosclerotic index and plaque areas of aortic roots, and attenuate macrophage-derived foam cell formation in ApoE^−/−^ mice by promoting macrophage autophagy via inhibiting the PI3K/Akt/mTORC1 signaling pathway (Zhou et al., 2019). Macrophage autophagy also plays an essential role in inflammatory response and oxidative stress. In ApoE-null mice, complete disruption of macrophage autophagy promotes hyperactivation of the macrophage inflammasome and excess interleukin-1*β* (IL-1*β*) production, thus increasing plaque instability (Razani et al., 2012). Basic autophagy can mitigate cell damage caused by oxidative stress by degrading intracellular oxidative damaged components and clearing dysfunctional mitochondria. It has been reported that macrophage ATG5 deficient Ldlr^−/−^ mice showed increased oxidative stress in the context of atherosclerosis, which expedited the development of atherosclerotic plaque (Liao et al., 2012). In addition, when macrophages are being cleared, macrophage autophagy seems to facilitate efferocytosis. Xianghai Liao et al. showed that in the mouse model of advanced atherosclerosis, blocking autophagy rendered macrophages more susceptible to die, worsened the recognition and removal of dead cells via efferocytes, and accelerated plaque necrosis (Liao et al., 2012). In conclusion, a moderate increase of macrophage autophagy can reduce intracellular lipid accumulation, decrease foam cell formation, inhibit inflammatory and oxidative stress response, and promote efferocytosis in atherosclerotic plaques, thereby delaying the progression of atherogenesis (Figure 2).

#### Pharmacological Intervention of Macrophage Autophagy in the Treatment of Atherosclerosis

Accumulating evidence has indicated that macrophage autophagy plays a protective role in the occurrence and development of atherosclerosis. It can inhibit inflammatory reaction, attenuate oxidative stress and promote cholesterol efflux, which provides a new research direction for the treatment of atherosclerosis (Go et al., 2013). mTOR inhibitors are the most studied autophagy inducers. Many reports have confirmed that mTOR inhibitors or their derivatives have protective effects on atherogenesis. Inhibition of mTOR with pharmacological agents such as rapamycin appears to inhibit atherosclerosis in plenty of different animal models (Pakala et al., 2005; Mueller et al., 2008). Sirtuin1 (Sirt1), belonging to the conservative sirtuin family, exerts protective effects via regulating autophagy as an essential regulator during the progression of atherosclerosis. In Baoxin Liu’s study, autophagy was upregulated by the Sirt1 activator resveratrol (RSV). When incubated with the appropriate dose of RSV, the efferocytosis of apoptotic RAW264.7 increased significantly, which alleviated inflammation in atherosclerotic plaques and avoided plaque rupture (Liu et al., 2014). Like Sirt1, Sirt6 also plays a crucial role in reducing plaque formation and promoting plaque stability via stimulating macrophage autophagy, and thereby revealing a novel target for therapeutic interventions (Wang et al., 2020). Additionally, Ismail Sergin et al. showed that therapeutical-related doses of trehalose can increase macrophage autophagy, TFEB (the master transcriptional regulator of autophagy–lysosomal biogenesis) and autophagy–lysosomal biogenesis both *in vitro* and *in vivo*, leading to reverse the dysfunction of macrophages in the plaque and mitigate atherosclerosis (Sergin et al., 2017). Therefore, inducing macrophage autophagy may be a potential therapeutic strategy for atherosclerosis.

### Effect of Macrophage Pyroptosis on Atherosclerosis

#### Pyroptosis and Its Main Characteristics

Pyroptosis, also known as inflammatory necrosis, is a new way of programmed cell death, which depends on the caspase family. Pyroptosis is accompanied by the maturation and release of inflammatory factors such as IL-1*β* and interleukin-18 (IL-18), and induces inflammatory cascade reaction. The pyroptosis signaling pathway can be divided into caspase-1-dependent classical pathways and caspase-4/5/11-dependent non-classical pathways. Pyroptosis is often induced by viral or bacterial infection and endogenous damage, which plays an essential role in antagonizing infection and endogenous danger signals. It is one of the innate immune defense mechanisms of hosts against intracellular pathogens infection (Miao et al., 2010).

Shao Feng’s team and Vishva M Dixit’s team discovered respectively in 2015 that caspase-1 and caspase-4/5/11 induced pyroptosis by cutting a protein called Gasdermin-D (GSDMD). After being cut by caspase-1 or caspase-4/5/11, GSDMD released its N-terminal domain which had the activity of binding to membrane lipids and drilling holes in the cell membrane, which led to changes in cell osmotic pressure and swelling until the final rupture of the cell membrane (Kayagaki et al., 2015; Shi et al., 2015). The pathway of pyroptosis is mainly regulated by inflammatory bodies. At present, the inflammatory bodies most closely related to atherosclerosis are mainly NLRP3 inflammatory bodies which are members of the NLR family. The NLR family’s main biological function is to strengthen the ability of the immune system to detect microbial infection, produce proinflammatory cytokines, and regulate immune and inflammatory response. In the classical pathway of pyroptosis, related stimulation leads to the activation of NLRP3 which then recruits and activates caspase-1. The activated caspase-1 cleaves and activates inflammatory cytokines such as IL-1β and IL-18, and cleaves the N-terminal sequence of GSDMD which binds to the membrane to produce membrane pores, resulting in pyroptosis.

#### The Effectiveness of Macrophage Pyroptosis on Atherosclerosis

**FIGURE 3:**
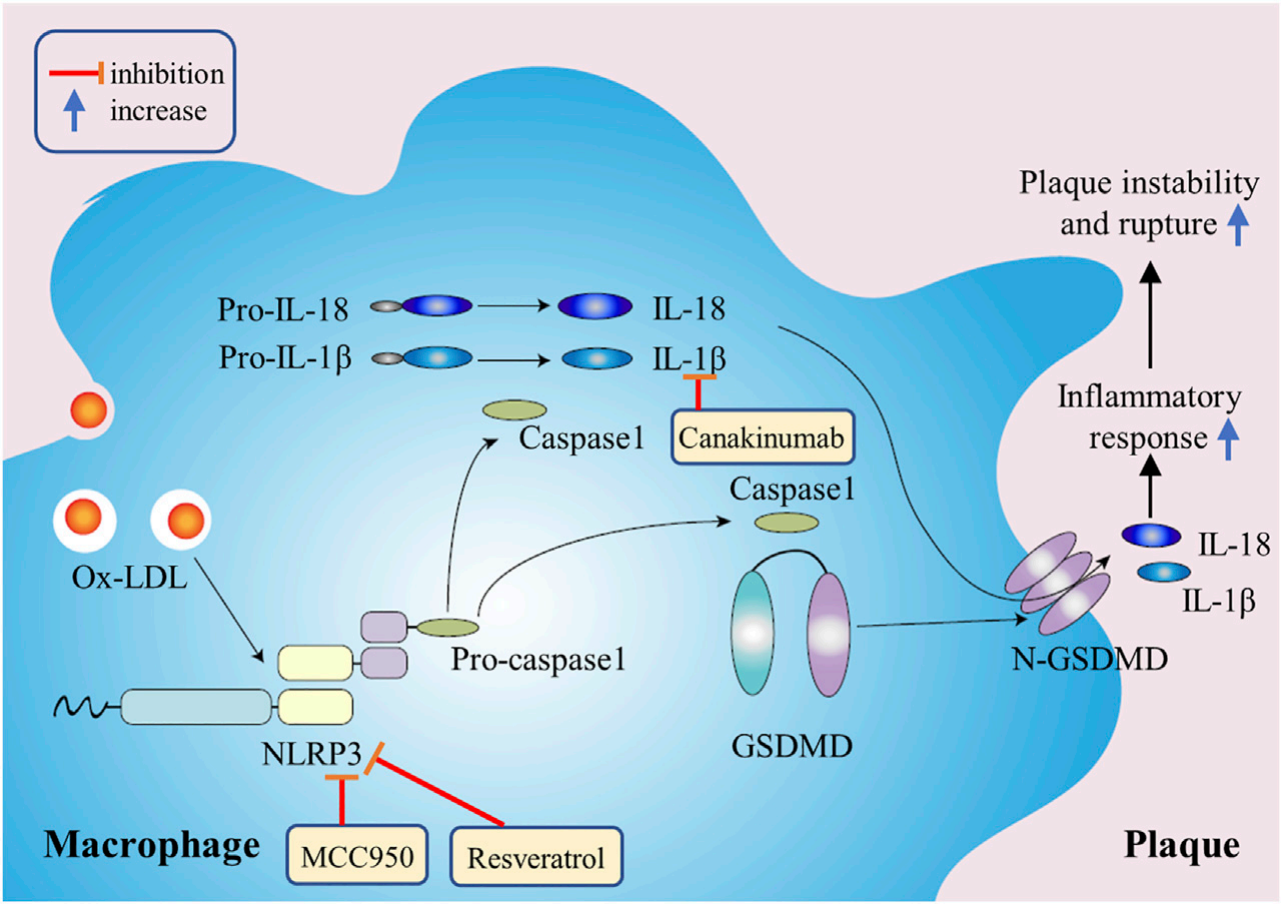
The mechanism of macrophage pyroptosis in promoting atherosclerosis. Ox-LDL in plaques will continuously activate NLRP3 inflammatory bodies and caspase-1, resulting in a continuous increase in the release of inflammatory factors, thus promoting plaque instability and rupture.

In recent years, a growing body of evidence suggests that macrophage pyroptosis plays a more significant role in the formation, rupture and immuno-inflammatory response of atherosclerotic vulnerable plaques than traditional ways of programmed cell death, such as apoptosis, autophagy and so on (Xu et al., 2018). Normal level of pyroptosis can cleave cells and release pathogens and inflammatory factors outside the cells, so as to recruit immune cells to remove pathogens and damaged cells, which is conducive to the maintenance of local homeostasis. However, in atherosclerosis, ox-LDL and cholesterol crystallization in the plaque will continuously activate NLRP3 inflammatory bodies and caspase-1, resulting in a continuous increase in the release of inflammatory factors, thus promoting the enhancement of inflammatory response in the atherosclerotic region and reducing the stability of the plaque (Hoseini et al., 2018) (Figure 3). It has been reported that NLRP3 inflammatory bodies are highly expressed in atherosclerotic areas, silencing ApoE^−/−^ mice NLRP3 gene can inhibit the expression of inflammatory factors and atherosclerotic progression, reduce the content of lipids and matrix metalloproteinases in plaques, increase collagen fibers, promote plaque stability, and then reduce the risk of atherosclerotic plaque rupture (Zheng et al., 2014). In atherosclerotic plaques, in addition to overactivation of NLRP3, researchers also discovered that the site of plaque rupture demonstrated a highly immunoreactivity to caspase-1 in macrophages, while caspase-3 staining was weak, suggesting that macrophage pyroptosis may play a more vital role in atherosclerotic lesions than apoptosis (Kolodgie et al., 2000). Furthermore, IL-1*β*, IL-18 and other pro-inflammatory cytokines produced and released by macrophages in atherosclerotic lesion are also closely associated with the development and stability of atherosclerotic plaques. IL-1*β* and IL-18 are predominantly derived from macrophages and are the main substrates of caspase-1. Hirokazu Kirii et al. reported that IL-1*β* deficiency reduced atherosclerotic lesions by about 33% in ApoE^−/−^ mice (Kirii et al., 2003). In short, the release of inflammatory factors enhances the inflammatory environment and increases the aggregation of macrophages, thus promoting the development and instability of plaques.

#### Pharmacological Intervention of Macrophage Pyroptosis in the Treatment of Atherosclerosis

Many studies have confirmed that macrophage pyroptosis plays a crucial role in the formation, rupture and immune inflammation of atherosclerotic plaques. Regulation of macrophage pyroptosis is expected to become a new strategy to stabilize vulnerable plaques (Xu et al., 2018).

Activated NLRP3 inflammatory bodies and IL-1*β* signaling pathway may be essential to the atherosclerotic lesion progression. NLRP3 inflammatory body selective inhibitor MCC950 can reduce macrophage aggregation and inflammatory factor levels, significantly inhibit atherosclerotic plaque progression and increase plaque stability in ApoE^−/−^ mice. MCC950 can also inhibit ox-LDL uptake, increase cholesterol efflux and inhibit the transformation of macrophages into foam cells (Chen et al., 2018). Canakinumab is a therapeutic monoclonal antibody targeting IL-1*β* with anti-inflammatory effects. The recurrence rate of cardiovascular events among atherosclerotic patients assigned to receive canakinumab was significantly lower than among those in the placebo group (Ridker et al., 2017). There were other researchers who used the effective ingredients of anti-inflammatory and detoxifying traditional Chinese medicine to carry out the research on the treatment of atherosclerosis. Misawa T et al. found that RSV was able to inhibit the assembly of NLRP3 and apoptosis-related spect-like protein containing a CARD (ASC) by inhibiting *a*-tubulin acetylation, and then inhibit the activation of NLRP3 inflammatory bodies and pyroptosis in mouse macrophages (Misawa et al., 2015). Berberine, an active component of coptis chinensis, can inhibit the activation of ROS-dependent NLRP3 inflammatory bodies and reduce the synthesis of pro-IL-1*β* by inhibiting NF-κB, so it can be used in the prevention and treatment of atherosclerosis (Ju et al., 2018).

### Effect of Macrophage Ferroptosis on Atherosclerosis

#### Ferroptosis

Ferroptosis is a new type of iron-dependent programmed cell death, which occurs through the lethal accumulation of ROS when glutathione (GSH)-dependent lipid peroxidation repair system is compromised (Stockwell et al., 2017). Ferroptosis is closely related to a variety of human diseases, such as cancers, cardiovascular diseases and degenerative diseases. The essence of ferroptosis is the depletion of glutathione, the decrease of glutathione peroxidase (GPXs) activity (the decrease of GPX4 activity is the most important), and the impaired antioxidant capacity of cells dependent on GPX4. Lipid oxides cannot be metabolized by GPX4-catalyzed glutathione reductase reaction, and then Fe^2+^ oxidizes lipids to produce ROS, which eventually leads to cell oxidative death, that is, ferroptosis. There is a significant difference between ferroptosis and other forms of programmed cell death. In cell morphology, the changes of ferroptosis are mainly the increase of mitochondrial membrane density and mitochondrial contraction, but not accompanied by the decrease of nuclear volume, chromatin condensation and so on (Dixon et al., 2012). In the aspect of biochemical metabolism, ferroptosis is characterized by the increase of intracellular lipid peroxidation, the increase of ROS, the breaking of oxidation-antioxidation balance, the loss of cell integrity and cell death, which can be inhibited by antioxidants and iron chelating agents (Li et al., 2020).

#### The Effectiveness of Macrophage Ferroptosis on Atherosclerosis

**FIGURE 4:**
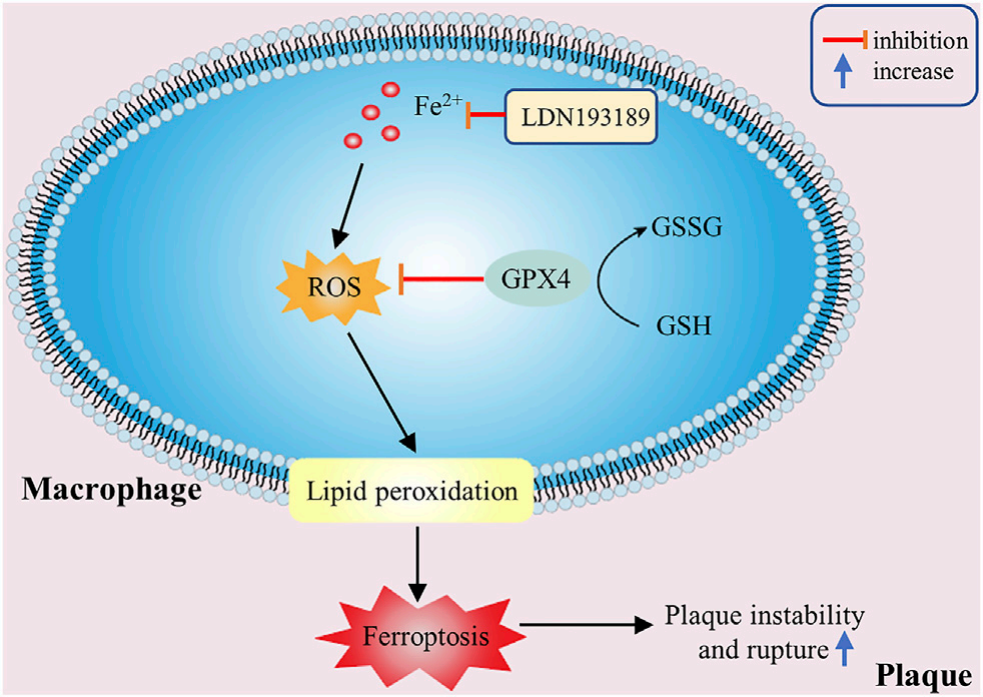
The effectiveness of macrophage ferroptosis on atherosclerosis. Excessive ROS accumulation or iron overload in macrophages can promote lipid peroxidation and other pathological processes, then cause macrophage ferroptosis and increase plaque instability.

Atherosclerosis is closely related to the changes of ROS and iron levels in the body. Excessive ROS accumulation or iron overload can promote cellular oxidative stress, lipid peroxidation and other pathological processes, and increase plaque instability (Figure 4). ROS accumulation in macrophages, lipid peroxidation, plaque hemorrhage and iron deposition are important characteristics of advanced atherosclerotic plaque, which indirectly indicates that ferroptosis may be involved in the development of atherosclerosis (Martinet et al., 2019). Besides, the increase of free iron can also accelerate inflammation and the formation of macrophage-derived foam cells (Hu et al., 2019). When Omar Saeed et al. used LDN 193189, a small molecular inhibitor of BMP signal transduction, to inhibit ferritin, thereby reducing macrophage intracellular iron levels, macrophages from LDN treated ApoE^−/−^ mice demonstrated increased lipid efflux and reduced foam cell formation (Saeed et al., 2012). The loss of the activity of GPX4, the key enzyme of ferroptosis, will also lead to the accumulation of a large number of lipid peroxides and ROS, which further affect the progress of atherosclerosis (Feng and Stockwell, 2018). And overexpression of GPX4 can reduce lipid peroxidation and inhibit plaque development in ApoE^−/−^ mice (Guo et al., 2008). These studies demonstrate that iron overload and lipid peroxidation play a significantly important role in the occurrence and development of atherosclerosis, and ferroptosis is closely related to atherogenesis. Therefore, removing excess iron and reducing ROS production may be new strategies for the treatment and prevention of atherosclerosis in the future.

### Effect of Macrophage Necroptosis on Atherosclerosis

#### Necroptosis

Necroptosis, known as programmed cell necrosis, is also one of the important ways of programmed cell death, which was first proposed by Degterev et al., in 2005 (Degterev et al., 2005). Necroptosis has typical morphological features of necrosis, such as increase of cell size, swelling of organelles and rupture of cell plasma membrane. In terms of physiological and biochemical characteristics, necroptosis produces a large number of ROS and pro-inflammatory factors, and often forms necrosomes in the process of necrosis. However, compared with the usual necrosis, necroptosis is an ordered necrosis with the unique signal pathway and regulated by specific signal molecules (Kroemer et al., 2009). In necroptosis, receptor-interacting proteins (RIP) are a class of important signal molecules regulating cell death or survival, especially receptor-interacting protein kinase 1 (RIPK1) and receptor-interacting protein kinase 3 (RIPK3) play a key regulatory role in necroptosis signal pathway, and its expression level can indicate the degree of necroptosis (Silke et al., 2015). The classical pathway of necroptosis depends on the binding of tumor necrosis factor-*α* (TNF-α) to membrane receptors, which transduces signals to RIPK1 and RIPK3, so that they can phosphorylate each other to obtain kinase activity, and then phosphorylate downstream mixed lineage kinase domain-like protein (MLKL). The activated MLKLs oligomerize and form selective ion channels, which eventually lead to the rupture of cell membrane and cell necroptosis. In addition, when apoptosis is inhibited, necroptosis can be used as an alternative way to mediate cell death.

#### The Effectiveness of Macrophage Necroptosis on Atherosclerosis

**FIGURE 5:**
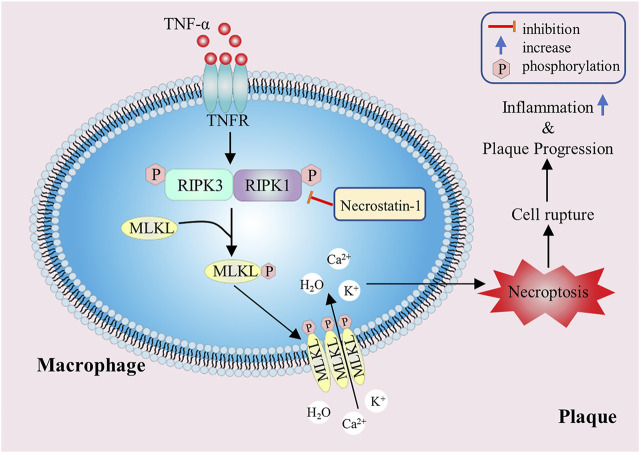
The promoting effect of macrophage necroptosis on atherosclerosis. The RIPK1 and RIPK3 play a key regulatory role in necroptosis, and they can phosphorylate downstream MLKL. The activated MLKLs form selective ion channels, which eventually lead to cell membrane rupture, inflammatory factors release and plaque instability.

The radiotracers labeling the necroptosis pathway were located in atherosclerotic plaques, which proved that necroptosis was involved in the pathological process of atherosclerosis (Leeper, 2016). Studies have shown that once programmed necrosis occurs, it will release a large number of inflammatory factors and chemokines, induce severe inflammatory reaction and promote the formation of atherosclerotic plaques (Galluzzi et al., 2017). Further study found that macrophage necroptosis can directly lead to the formation of atherosclerotic necrotic core and plaque instability (Kavurma et al., 2017) (Figure 5). Lingjun Meng et al. demonstrated that in RIPK3 knockout atherosclerotic mouse models, the levels of inflammatory factors such as IL-6 and IL-1β were significantly decreased, and the size of atherosclerotic plaque was reduced (Meng et al., 2016). Juan Lin et al. also found that in Ldlr^−/−^ mice, the area of advanced atherosclerotic plaques in RIPK3 knockout mice was significantly reduced, and the number of programmed necrotic macrophages in the lesion was less than that in wild mice, but there was no significant difference in the number of apoptotic cells, suggesting that RIPK3-dependent necroptosis was closely related to the development of advanced atherosclerosis (Lin et al., 2013). Similar results were observed in RIPK1 knockout mice. According to Denuja Karunakaran’s research results, knockout of RIPK1 in macrophages *in vitro* and *in vivo* can reduce inflammatory gene expression stimulated by TNF-α, inhibit nuclear factor κ-B (NF-κB) activity, markedly reduce atherosclerotic lesions, and have no effect on plasma cholesterol and body weight (Karunakaran et al., 2021). In ApoE^−/−^ mice, necroptosis inhibitor necrostatin-1 (Nec-1) can inhibit the formation of RIPK1 and RIPK3 complex, then block the occurrence of macrophage necroptosis and mitigate the inflammatory response, thus inducing significant reduction in atherosclerotic lesions (Karunakaran et al., 2016). At present, Nec-1 has attracted wide attention as a new target for diagnosis, intervention and treatment of patients with atherosclerosis. Hence, inhibiting macrophage necroptosis in atherosclerotic lesions may have the effect of reducing intravascular load and stabilizing atherosclerotic plaques. Moreover, drugs or inhibitors targeting RIPK1 and RIPK3 can be designed to slow down atherosclerosis progression by mitigating macrophage necroptosis.

## Conclusion

Macrophage programmed cell death is closely related to the formation and instability of atherosclerotic plaques, and it plays a vital role in the occurrence and development of atherosclerosis. This suggests that it can be a potential therapeutic target for vascular disease. Although the relationship between macrophage programmed cell death and atherosclerosis has been gradually recognized, many of the specific mechanisms are still not clear. In particular, the microenvironment in atherosclerotic plaques is complex, and a variety of inducing factors of macrophage death exist at the same time, so there are many ways of macrophage death in atherosclerotic plaques simultaneously. Moreover, the complicated relationship between diverse types of macrophage death requires us to target multiple types at the same time.

In addition, various death modes of macrophages have diverse effects on atherogenesis in different stages, so strategies should be adopted for different stages and modes of atherosclerosis. In the early stage of atherosclerosis, the efferocytosis of phagocytes is efficient, consequently, measures should be taken to enhance the specific apoptosis of macrophages, so as to reduce the production of macrophage-derived foam cells and inflammatory reaction, then mitigate disease development and stabilize plaques. In the late stage of efferocytosis dysfunction, macrophage apoptosis should be inhibited to prevent a large number of apoptotic cells from not being absorbed and cleared in time, from resulting in secondary cell necrosis and leading to inflammation and plaque rupture. Under controlled conditions, macrophage autophagy can promote plaque stability and delay disease development, so increasing macrophage autophagy can prevent the further development of plaques. But excessive autophagy which may cause apoptosis will exert harmful functions in atherosclerosis. Furthermore, the pyroptosis of macrophages that cause inflammation and ferroptosis that aggravate oxidative stress should be inhibited throughout the development of atherosclerosis. Macrophage necroptosis within plaques also secretes large amounts of inflammatory cytokines and ROS, so it should be inhibited as well. In summary, although some progress has been made in the study of programmed macrophage death in atherosclerotic plaques, the specific mechanism, signal transduction pathway and significance in clinical treatment have not been clearly elucidated. Further research is needed to solve the existing obstacles.
